# Response to: Comment on “Analysis of Microarray-Identified Genes and MicroRNAs Associated with Idiopathic Pulmonary Fibrosis”

**DOI:** 10.1155/2019/3192089

**Published:** 2019-03-04

**Authors:** Lichao Fan, Xiaoting Yu, Ziling Huang, Shaoqiang Zheng, Yongxin Zhou, Hanjing Lv, Yu Zeng, Jin-Fu Xu, Xuyou Zhu, Xianghua Yi

**Affiliations:** ^1^Department of Pathology, Tongji Hospital, Tongji University School of Medicine, Shanghai 200065, China; ^2^Department of Respiratory and Critical Care Medicine, Shanghai Pulmonary Hospital, Tongji University School of Medicine, Shanghai 200443, China; ^3^Department of Radiology, Tongji Hospital, Tongji University School of Medicine, Shanghai 200065, China; ^4^Department of Thoracic-Cardiovascular Surgery, Tongji Hospital, Tongji University School of Medicine, Shanghai 200065, China; ^5^Department of Respiratory Medicine, Shanghai Tongji Hospital, Tongji University School of Medicine, Shanghai 200065, China

Recently, we were questioned [1] about the quality assessment of the raw data which we used in the article “Analysis of Microarray-Identified Genes and MicroRNAs Associated with Idiopathic Pulmonary Fibrosis” published in 14 May 2017 [2]. Aiming at this issue, we asked for the original qualified data of GSE32537 and GSE32538 from the previous authors in Yang's group [3] and also tested the other samples from GSE53845 and GSE10667. All the qualified control results for GSE32537 (Figure 1(a)–1(c)), GSE32538 (Figure 1(d)), GSE53845 (Table 1), and GSE10667 (Table 2) are listed as follows. No unqualified samples were found.

Partek and RLE plot were provided by Yang's group. There is no unqualified sample in GSE32537 (Figure 1(a)–1(c)) and GSE32538 (Figure 1(a)). Partek software has been cited in over six thousand peer-reviewed articles published in scientific journals such as the prestigious *New England Journal of Medicine*, *Cell*, and *Nature* [4, 5]. Topics include drug research, human genetics, and disease relationships, causes, diagnosis, and treatments (http://www.partek.com/publications). Also, it is recommended by the NIH (https://ostr.cancer.gov/btep/software/partek).

According to the samples from GSE10667 (Table 1) and GSE53845 (Table 2), because the data are obtained in the Agilent chip platform, they can be qualified using CV and check ratio (http://www.chem.agilent.com/cag/bsp/products/1color/OnecolorPerformanceposter_finaldraft.pdf), and we found no unqualified samples.

## Figures and Tables

**Figure 1:**
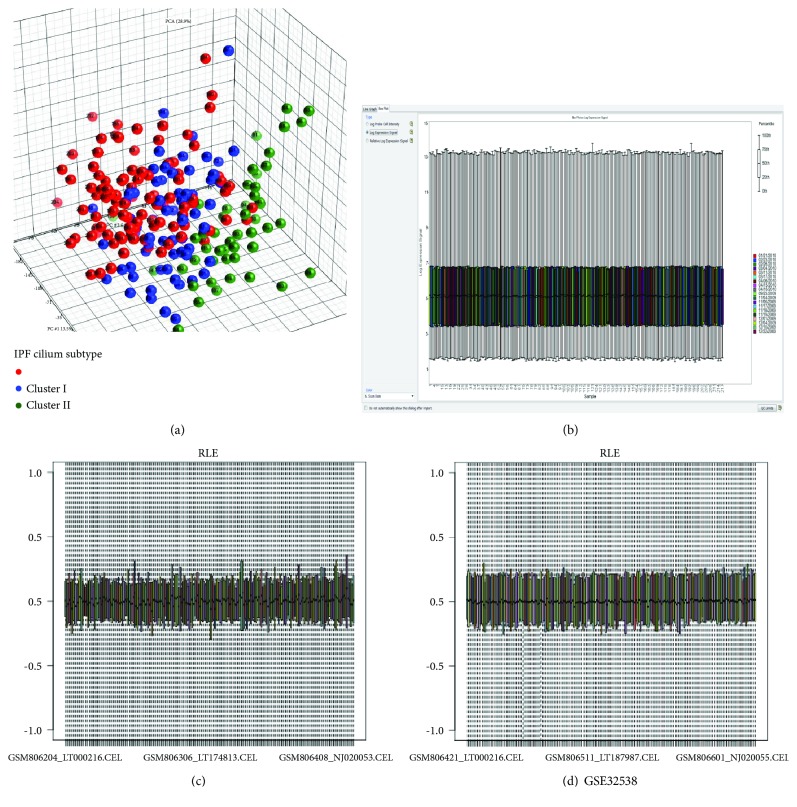


**Table 1:** GSE10667: no unqualified sample.

Sample	CV	Check ratio (%)
GSM269751	4.52039	53.47336341
GSM269752	4.41297	52.33499656
GSM269753	3.81111	62.98664436
GSM269754	3.09607	73.82951388
GSM269755	4.31694	68.26615674
GSM269756	4.48302	68.00322687
GSM269757	3.28292	72.78078222
GSM269758	5.05705	60.387224
GSM269759	4.8888	78.5293854
GSM269760	4.37522	65.56514984
GSM269761	4.71739	71.8186979
GSM269762	4.73779	71.97406555
GSM269763	5.82171	65.89978786
GSM269764	5.3492	70.23215513
GSM269765	5.62496	67.63572261
GSM269766	4.30253	68.77408946
GSM269767	4.07308	69.87660223
GSM269768	4.16314	72.49096179
GSM269769	3.56498	71.66631809
GSM269770	4.08422	77.92584182
GSM269771	4.06119	71.89638173
GSM269772	5.20147	75.22483492
GSM269773	4.47771	70.84167439
GSM269774	3.62776	74.70793869
GSM269775	4.83568	76.92491559
GSM269776	3.11648	81.40667483
GSM269777	4.33606	76.76954794
GSM269778	5.75702	59.62532493
GSM269779	3.90341	73.55463265
GSM269780	5.763	56.18034599
GSM269781	3.74164	67.74328483
GSM269782	4.05046	72.10851833
GSM269783	3.50132	79.10305058
GSM269784	3.7863	76.78747498
GSM269785	5.56487	70.80582031
GSM269786	4.50389	69.05793421
GSM373881	5.41032	65.92667842
GSM373882	4.93834	66.57503959
GSM373883	5.85297	64.23556127
GSM373884	4.38777	56.10863784
GSM373885	5.66725	70.55782963
GSM373886	5.9859	59.38032209
GSM373887	4.98283	66.22546237
GSM373888	4.16159	68.11975261
ControlType.GSM269749	4.63445	68.07194717
ControlType.GSM269750	5.14763	66.67065045

**Table 2:** GSE53845: no unqualified sample.

Sample	CV	Check ratio (%)
GSM1302038_Agilent_251485063787_S01_GE2_107_Sep09_1_1	3.34	80.89
GSM1302039_Agilent_251485063802_S01_GE2_107_Sep09_1_2	2.79	84.28
GSM1302040_Agilent_251485053914_S01_GE2_107_Sep09_1_3	3.49	75.42
GSM1302041_Agilent_251485063803_S01_GE2_107_Sep09_1_1	3.28	85.06
GSM1302042_Agilent_251485053914_S01_GE2_107_Sep09_1_4	3.82	76.68
GSM1302043_Agilent_251485063886_S01_GE2_107_Sep09_1_1	5.60	79.04
GSM1302044_Agilent_251485053915_S01_GE2_107_Sep09_1_1	2.96	67.53
GSM1302045_Agilent_251485063787_S01_GE2_107_Sep09_1_4	3.48	81.01
GSM1302046_Agilent_251485063886_S01_GE2_107_Sep09_1_3	2.83	82.84
GSM1302047_Agilent_251485063785_S01_GE2_107_Sep09_1_2	3.24	82.42
GSM1302048_Agilent_251485053915_S01_GE2_107_Sep09_1_3	3.14	79.97
GSM1302049_Agilent_251485063785_S01_GE2_107_Sep09_1_1	8.70	80.38
GSM1302050_Agilent_251485063801_S01_GE2_107_Sep09_1_4	3.66	71.63
GSM1302051_Agilent_251485063803_S01_GE2_107_Sep09_1_4	2.95	77.05
GSM1302052_Agilent_251485063802_S01_GE2_107_Sep09_1_1	3.27	79.84
GSM1302053_Agilent_251485063803_S01_GE2_107_Sep09_1_3	2.78	81.38
GSM1302054_Agilent_251485053915_S01_GE2_107_Sep09_1_4	2.68	84.37
GSM1302055_Agilent_251485063783_S01_GE2_107_Sep09_1_1	2.75	87.23
GSM1302056_Agilent_251485063799_S01_GE2_107_Sep09_1_1	3.06	72.31
GSM1302057_Agilent_251485053914_S01_GE2_107_Sep09_1_2	3.11	80.21
GSM1302058_Agilent_251485063799_S01_GE2_107_Sep09_1_3	2.74	86.21
GSM1302059_Agilent_251485063801_S01_GE2_107_Sep09_1_3	2.80	81.95
GSM1302060_Agilent_251485063799_S01_GE2_107_Sep09_1_2	3.29	84.60
GSM1302061_Agilent_251485063886_S01_GE2_107_Sep09_1_2	3.07	84.19
GSM1302062_Agilent_251485063783_S01_GE2_107_Sep09_1_4	2.91	82.97
GSM1302063_Agilent_251485063803_S01_GE2_107_Sep09_1_2	2.82	85.06
GSM1302064_Agilent_251485063787_S01_GE2_107_Sep09_1_2	3.20	83.71
GSM1302065_Agilent_251485063799_S01_GE2_107_Sep09_1_4	3.62	76.77
GSM1302066_Agilent_251485063801_S01_GE2_107_Sep09_1_2	3.54	71.39
GSM1302067_Agilent_251485063801_S01_GE2_107_Sep09_1_1	3.54	78.49
GSM1302068_Agilent_251485063784_S01_GE2_107_Sep09_1_1	2.91	83.60
GSM1302069_Agilent_251485063802_S01_GE2_107_Sep09_1_3	3.28	69.97
GSM1302070_Agilent_251485063800_S01_GE2_107_Sep09_1_3	3.18	82.97
GSM1302071_Agilent_251485063783_S01_GE2_107_Sep09_1_3	3.95	86.68
GSM1302072_Agilent_251485063802_S01_GE2_107_Sep09_1_4	3.08	89.20
GSM1302073_Agilent_251485063784_S01_GE2_107_Sep09_1_3	3.69	78.97
GSM1302074_Agilent_251485063784_S01_GE2_107_Sep09_1_4	2.60	86.89
GSM1302075_Agilent_251485063787_S01_GE2_107_Sep09_1_3	2.70	87.34
GSM1302076_Agilent_251485063785_S01_GE2_107_Sep09_1_3	2.91	79.15
GSM1302077_Agilent_251485053915_S01_GE2_107_Sep09_1_2	2.83	85.94
GSM1302078_Agilent_251485063800_S01_GE2_107_Sep09_1_1	3.19	84.51
GSM1302079_Agilent_251485063783_S01_GE2_107_Sep09_1_2	3.23	85.24
GSM1302080_Agilent_251485053914_S01_GE2_107_Sep09_1_1	3.46	81.72
GSM1302033_Agilent_251485063785_S01_GE2_107_Sep09_1_4	2.92	83.53
GSM1302034_Agilent_251485063800_S01_GE2_107_Sep09_1_2	4.09	82.70
GSM1302035_Agilent_251485063784_S01_GE2_107_Sep09_1_2	3.00	86.25
GSM1302036_Agilent_251485063800_S01_GE2_107_Sep09_1_4	4.20	81.71
GSM1302037_Agilent_251485063886_S01_GE2_107_Sep09_1_4	2.80	82.54

## References

[1] Li C., Wang S., Che L., Wang X., Xu Y. (2018). Comment on “Analysis of microarray-identified genes and microRNAs associated with idiopathic pulmonary fibrosis”. *Mediators of Inflammation*.

[2] Fan L., Yu X., Huang Z. (2017). Analysis of microarray-identified genes and microRNAs associated with idiopathic pulmonary fibrosis. *Mediators of Inflammation*.

[3] Yang I. V., Coldren C. D., Leach S. M. (2013). Expression of cilium-associated genes defines novel molecular subtypes of idiopathic pulmonary fibrosis. *Thorax*.

[4] Kalin J. H., Wu M., Gomez A. V. (2018). Targeting the CoREST complex with dual histone deacetylase and demethylase inhibitors. *Nature Communications*.

[5] Christ A., Günther P., Lauterbach M. A. R. (2018). Western diet triggers NLRP3-dependent innate immune reprogramming. *Cell*.

